# Stone Characteristics and Surgical Treatment Patterns Among Patients With Urolithiasis at a Brazilian Referral Hospital: A Retrospective Observational Study

**DOI:** 10.7759/cureus.117863

**Published:** 2026-10-13

**Authors:** Arthur B Bernardes, Lucas S Pontes, Ruan P Melotti, Alexander H Casini

**Affiliations:** 1 School of Medicine, Escola Superior de Ciências da Santa Casa de Misericórdia de Vitória, Vitória, BRA; 2 Urology, Escola Superior de Ciências da Santa Casa de Misericórdia de Vitória, Vitória, BRA

**Keywords:** endourology, flexible ureterorenoscopy, percutaneous nephrolithotomy, rigid ureteroscopy, stone location, stone size, ureteroscopy, urolithiasis

## Abstract

Background: Urolithiasis is a common and recurrent disorder that can cause obstruction, infection, renal impairment, repeated emergency care, and the need for surgical intervention. Real-world treatment patterns may help referral centers understand case mix and plan endourological resources.

Objective: To characterize the institutional surgical case mix of patients undergoing operative treatment for urolithiasis at a Brazilian nonprofit teaching hospital. Secondary exploratory analyses assessed the associations of stone location and stone size with the primary surgical procedure performed.

Methods: This retrospective observational study included 624 unique patients aged 16 years or older who underwent rigid ureteroscopy, flexible ureterorenoscopy, or percutaneous nephrolithotomy between June 1 and December 31, 2025. Demographic, stone-related, operative, anesthetic, and ureteral stent variables were extracted from institutional records. Three secondary exploratory contingency analyses evaluated procedure versus stone location, procedure versus stone size, and stone location versus stone size using Pearson's chi-square tests; Cramer's V quantified effect size. Analyses involving missing stone size or location used complete cases.

Results: Men accounted for 361 patients (57.9%), and mean age was 46.6 ± 13.8 years. Flexible ureterorenoscopy was the most frequent primary procedure (436; 69.9%), followed by rigid ureteroscopy (147; 23.6%) and percutaneous nephrolithotomy (41; 6.6%). Spinal anesthesia, alone or with sedation, was used in 559 patients (89.6%), and a double-J stent was used in 448 (71.8%). Procedure distribution varied markedly according to stone location (χ^2^(4) = 1108.77, p < 0.001; Cramer's V = 0.946; n = 619) and stone size (χ^2^(6) = 301.23, p < 0.001; Cramer's V = 0.505; n = 591). Stone size and location were also associated (χ^2^(6) = 323.63, p < 0.001; Cramer's V = 0.525; n = 588).

Conclusions: Surgical care was dominated by minimally invasive endourological procedures. Procedure distribution varied markedly according to stone location and size. These findings describe the operative case mix of a Brazilian referral hospital and may support service planning and future prospective quality studies.

## Introduction

Urolithiasis is among the most common urological disorders and remains clinically important because of recurrence, acute pain, obstruction, infection, and repeated use of health services. A 2025 meta-analysis of 22 studies involving more than 1.2 million participants estimated a pooled global prevalence of approximately 11%, although estimates varied substantially according to diagnostic method, sampling strategy, sex, and geographic region [1]. Contemporary epidemiologic reviews similarly show a persistent male predominance in many populations, together with a narrowing sex gap and marked regional heterogeneity [2].

The burden of stone disease extends beyond an isolated episode of renal colic. Recurrent stones may require repeated imaging, emergency evaluation, analgesia, drainage, and definitive intervention, and nephrolithiasis is associated with metabolic disorders that include obesity, diabetes mellitus, and other systemic conditions [2,3]. In Brazil, national public-hospital data have documented a sustained burden of stone-related admissions across age groups, while the most recent Brazilian Society of Nephrology guideline emphasizes the need for structured evaluation, prevention, and follow-up [3,4]. These observations support examining how referral centers deliver surgical care for stone disease.

In Brazil, however, the availability of endourological resources varies across treatment settings. In a national survey of 1,267 Brazilian urologists, rigid ureteroscopy was widely available, whereas access to flexible ureteroscopy and percutaneous nephrolithotomy was less uniform; equipment, infrastructure, and practical experience were reported as limiting factors [5]. These national data characterize resource availability but do not describe the patient-level surgical case mix within an individual referral hospital, including how stone size and anatomic location are distributed across procedures. Institutional data can therefore provide complementary information on operative workload and patterns of surgical care.

When active treatment is required, stone size and anatomic location are major considerations, together with obstruction, infection, urinary tract anatomy, renal function, patient characteristics, available technology, and surgeon expertise. Contemporary guideline frameworks support individualized selection among ureteroscopy, shock-wave lithotripsy, and percutaneous nephrolithotomy according to these factors [6-9]. These recommendations provide clinical context for interpreting procedural patterns but were not used in the present study to assess guideline adherence or comparative effectiveness.

Hospital Santa Casa de Misericórdia de Vitória is a nonprofit teaching hospital in Vitória, Espírito Santo, southeastern Brazil, and serves as a regional referral center for urological care. The primary objective of this study was to characterize the demographic, geographic, stone-related, operative, anesthetic, and ureteral-stent profile of patients treated surgically for urolithiasis at this institution. Secondary exploratory analyses quantified the associations of stone location and stone size with the primary surgical procedure performed. The study was designed to describe real-world surgical case mix and treatment patterns rather than to compare treatment effectiveness, assess adherence to guidelines, or infer causal treatment selection.

## Materials and methods

Study design and setting

This was a retrospective observational study based on medical record and institutional surgical data review. It was conducted at Hospital Santa Casa de Misericórdia de Vitória, a nonprofit teaching hospital in Vitória, Espírito Santo, Brazil. The study covered surgical care delivered from June 1 through December 31, 2025. Data sources included electronic medical records, operating room records, referral and counter-referral forms, and institutional urological regulation records. Data were extracted using a standardized collection instrument and organized in an electronic study table. Reporting was structured in accordance with the Strengthening the Reporting of Observational Studies in Epidemiology (STROBE) statement [10].

Participants and eligibility

The study population comprised patients aged 16 years or older who underwent operative treatment for urinary stone disease during the study period. A census sampling strategy was used, with all eligible cases identified in the period included. Patients were eligible when rigid ureteroscopy, flexible ureterorenoscopy, or percutaneous nephrolithotomy was performed for urinary calculi and documented in the hospital system. Records were excluded when no surgical procedure for urolithiasis was performed, when a planned procedure was canceled, or when the record was duplicated. The final cohort comprised 624 patients.

The analytic unit was the patient, and each patient contributed a single observation to the patient-level analyses. Twenty-two patients underwent more than one procedure during the same anesthetic surgical session. Seventeen underwent bilateral flexible ureterorenoscopy for bilateral stone disease and were classified once as flexible ureterorenoscopy. Five underwent percutaneous nephrolithotomy with complementary flexible ureterorenoscopy during the same session and were classified once as percutaneous nephrolithotomy for the patient-level procedure analyses. Additional procedures did not generate duplicate patient observations.

Variables and definitions

Collected variables included sex, age, municipality of origin, primary surgical procedure, anesthetic technique, double-J ureteral stent use, stone laterality, stone location, stone size, and whether more than one procedure was performed during the same operative session. Procedures were categorized as rigid ureteroscopy, flexible ureterorenoscopy, and percutaneous nephrolithotomy. Primary procedure categories were mutually exclusive at the patient level. Bilateral flexible ureterorenoscopy was categorized as a single flexible ureterorenoscopy observation. When percutaneous nephrolithotomy and complementary flexible ureterorenoscopy were performed during the same session, the case was classified under percutaneous nephrolithotomy only.

Stone location was classified as proximal ureter, distal ureter, intrarenal, or undocumented. Laterality was classified as right, left, or bilateral. Stone size was categorized exactly as recorded in the final study table: 0-5 mm, 5.1-10 mm, 10.1-20 mm, or >20 mm. For bilateral stone disease, the larger stone was used for stone size classification in order to preserve one observation per patient. Records without documented size were retained in overall descriptive analyses and excluded only from analyses requiring stone size.

Statistical analysis

Continuous variables were summarized using mean and standard deviation and, where available, median, interquartile range, minimum, and maximum. Categorical variables were summarized using counts and percentages. Three secondary exploratory contingency analyses quantified relationships already evident in the descriptive dataset: procedure versus stone location, procedure versus stone size, and stone location versus stone size. Pearson's chi-square tests assessed association, and Cramér's V quantified effect size. For each analysis, the chi-square statistic, degrees of freedom, p-value, and Cramér’s V were reported. Complete-case analysis was used for the variables included in each comparison, with no imputation. Expected cell counts were reviewed for each contingency table; no expected count was <1, and fewer than 20% of cells had expected counts <5. Tests were two-sided, and p < 0.05 was considered statistically significant. Calculations were performed in Python 3.12 using SciPy 1.17.0 (Python Software Foundation, Fredericksburg, VA, USA).

Because these analyses were exploratory and addressed related descriptive questions, interpretation emphasized the magnitude and pattern of association, including Cramer's V, rather than p-values alone; no causal or confirmatory inference was intended. A multivariable model was not attempted because clinically important potential confounders, including detailed stone burden, collecting system anatomy, infection status, comorbidity burden, and surgeon-level factors, were not systematically available.

Ethical considerations

The project was approved by the Research Ethics Committee of Escola Superior de Ciências da Santa Casa de Misericórdia de Vitória (CEP/EMESCAM), CAAE 97679926.7.0000.5065, approval number 8.425.379, issued May 12, 2026. The ethics committee waived the requirement for individual informed consent because the study was retrospective and based on information previously recorded in medical records. Data used for analysis were anonymized, access was restricted to the research team, and confidentiality and privacy were preserved.

## Results

A total of 624 patients underwent surgical treatment for urolithiasis during the seven-month study period. The largest numbers came from Vitória (143; 22.9%), Serra (138; 22.1%), Vila Velha (69; 11.1%), and Cariacica (68; 10.9%). Together, these four municipalities accounted for 418 patients (67.0%). Patients also came from multiple municipalities outside the metropolitan area, consistent with the regional referral function of the service. Men represented 361 patients (57.9%) and women 263 (42.1%). Mean age was 46.6 years (SD 13.8), median age was 46 years, the interquartile range was 36-56 years, and the observed age range was 16-88 years.

Flexible ureterorenoscopy was the most frequent procedure, performed in 436 patients (69.9%), followed by rigid ureteroscopy in 147 (23.6%) and percutaneous nephrolithotomy in 41 (6.6%). Ureteroscopic procedures accounted for 583 of 624 patients (93.4%). Mean age by procedure was 44.7 years for rigid ureteroscopy, 47.0 years for flexible ureterorenoscopy, and 49.3 years for percutaneous nephrolithotomy. These group means are descriptive and were not interpreted as evidence that age influenced procedure selection.

Spinal anesthesia with sedation was used in 321 patients (51.4%), spinal anesthesia alone in 238 (38.1%), and general anesthesia in 65 (10.4%). Thus, spinal anesthesia, with or without sedation, was used in 559 patients (89.6%). A double-J ureteral stent was used in 448 patients (71.8%). Stone laterality was right-sided in 338 patients (54.2%), left-sided in 269 (43.1%), and bilateral in 17 (2.7%). Twenty-two patients (3.5%) underwent more than one procedure during the same operative session. Seventeen underwent bilateral flexible ureterorenoscopy, and five underwent percutaneous nephrolithotomy with complementary flexible ureterorenoscopy. Each of these patients remained a single patient-level observation. These demographic, operative, anesthetic, stent, laterality, and stone characteristics are summarized in Table 1.

**Table 1:** Demographic, operative, and stone-related characteristics of the cohort (N = 624) Primary procedure counts are patient-level and mutually exclusive. Of the 22 patients who underwent more than one procedure during the same operative session, 17 underwent bilateral flexible ureterorenoscopy and were counted once as flexible ureterorenoscopy, while five underwent percutaneous nephrolithotomy with complementary flexible ureterorenoscopy and were counted once as percutaneous nephrolithotomy.

Characteristic	Category	n (%) or summary
Age, years	Mean ± SD	46.6 ± 13.8
	Median (IQR)	46 (36-56)
	Range	16-88
Sex	Male	361 (57.9)
	Female	263 (42.1)
Primary procedure	Flexible ureterorenoscopy	436 (69.9)
	Rigid ureteroscopy	147 (23.6)
	Percutaneous nephrolithotomy	41 (6.6)
Anesthesia	Spinal + sedation	321 (51.4)
	Spinal alone	238 (38.1)
	General	65 (10.4)
Double-J stent	Yes	448 (71.8)
	No	176 (28.2)
Laterality	Right	338 (54.2)
	Left	269 (43.1)
	Bilateral	17 (2.7)
Multiple procedures in same operative session	Yes	22 (3.5)
Stone location	Proximal ureter	417 (66.8)
	Distal ureter	160 (25.6)
	Intrarenal	42 (6.7)
	Undocumented	5 (0.8)
Stone size	0-5 mm	53 (8.5)
	5.1-10 mm	227 (36.4)
	10.1-20 mm	256 (41.0)
	>20 mm	55 (8.8)
	Undocumented	33 (5.3)

Procedure according to stone location

Procedure distribution varied markedly according to stone location. Among 417 patients with proximal ureteral stones, 413 (99.0%) underwent flexible ureterorenoscopy and four (1.0%) underwent rigid ureteroscopy. Among 160 patients with distal ureteral stones, 142 (88.8%) underwent rigid ureteroscopy, and 18 (11.3%) underwent flexible ureterorenoscopy. Among 42 patients with intrarenal stones, 41 (97.6%) underwent percutaneous nephrolithotomy and one (2.4%) underwent flexible ureterorenoscopy. Five records with undocumented location were excluded from this comparison.

A Pearson's chi-square test showed a strong association between stone location and procedure (χ^2^(4) = 1108.77, p < 0.001; Cramér’s V = 0.946; n = 619 complete records), as shown in Table 2.

**Table 2:** Primary procedure according to stone location among complete cases Percentages are within each stone-location category. Pearson's chi-square test: χ^2^(4) = 1108.77, p < 0.001; Cramér’s V = 0.946. Five records with undocumented location were excluded. Each patient contributed one mutually exclusive procedure category. Bilateral flexible ureterorenoscopy was counted once as flexible ureterorenoscopy; cases of percutaneous nephrolithotomy with complementary flexible ureterorenoscopy were classified as percutaneous nephrolithotomy.

Stone location	Rigid ureteroscopy, n (%)	Flexible ureterorenoscopy, n (%)	Percutaneous nephrolithotomy, n (%)	Total
Proximal ureter	4 (1.0)	413 (99.0)	0 (0.0)	417
Distal ureter	142 (88.8)	18 (11.3)	0 (0.0)	160
Intrarenal	0 (0.0)	1 (2.4)	41 (97.6)	42

Procedure according to stone size

Procedure distribution also varied by stone size. Ureteroscopic approaches predominated in all categories up to 20 mm. Among stones measuring 0-5 mm, flexible ureterorenoscopy accounted for 34 of 53 procedures (64.2%). In the 5.1-10 mm and 10.1-20 mm categories, flexible ureterorenoscopy accounted for 158 of 227 procedures (69.6%) and 200 of 256 (78.1%), respectively. In contrast, among stones >20 mm, percutaneous nephrolithotomy was the most frequent procedure, accounting for 34 of 55 cases (61.8%).

A Pearson's chi-square test showed a strong association between stone size and procedure (χ^2^(6) = 301.23, p < 0.001; Cramér’s V = 0.505; n = 591 complete records), as shown in Table 3. Thirty-three patients without documented stone size were excluded from this comparison.

**Table 3:** Primary procedure according to stone size among complete cases (n = 591) Percentages are within each stone-size category. Pearson's chi-square test: χ^2^(6) = 301.23, p < 0.001; Cramér’s V = 0.505. Thirty-three records with undocumented stone size were excluded. Each patient contributed one mutually exclusive procedure category. In bilateral stone disease, the larger stone was used for stone-size classification. Cases of percutaneous nephrolithotomy with complementary flexible ureterorenoscopy were classified as percutaneous nephrolithotomy.

Stone size	Rigid ureteroscopy, n (%)	Flexible ureterorenoscopy, n (%)	Percutaneous nephrolithotomy, n (%)	Total
0-5 mm	19 (35.8)	34 (64.2)	0 (0.0)	53
5.1-10 mm	68 (30.0)	158 (69.6)	1 (0.4)	227
10.1-20 mm	51 (19.9)	200 (78.1)	5 (2.0)	256
>20 mm	5 (9.1)	16 (29.1)	34 (61.8)	55

Relationship between stone size and location

Stone size also differed by anatomic location. Among proximal ureteral stones with documented size, 350 of 393 (89.1%) were in the 5.1-10 mm or 10.1-20 mm categories. Among distal ureteral stones with documented size, 127 of 155 (81.9%) were in these two categories. Intrarenal stones showed a different profile: 34 of 40 patients (85.0%) with complete size data had stones >20 mm.

Among the 588 patients with complete data for stone location and stone size, a Pearson's chi-square test showed a strong association between these variables (χ^2^(6) = 323.63, p < 0.001; Cramér’s V = 0.525), as shown in Table 4.

**Table 4:** Stone size according to stone location among complete cases for both variables (n = 588) Percentages are within each stone-location category. Pearson's chi-square test: χ^2^(6) = 323.63, p < 0.001; Cramér’s V = 0.525. In bilateral stone disease, the larger stone was used for stone-size classification.

Stone location	0-5 mm	5.1-10 mm	10.1-20 mm	>20 mm	Total
Proximal ureter	30 (7.6)	153 (38.9)	197 (50.1)	13 (3.3)	393
Distal ureter	23 (14.8)	73 (47.1)	54 (34.8)	5 (3.2)	155
Intrarenal	0 (0.0)	1 (2.5)	5 (12.5)	34 (85.0)	40

## Discussion

This study provides a patient-level description of surgical urolithiasis care in 624 unique patients treated at a Brazilian nonprofit referral hospital. The cohort was concentrated in middle age, showed a modest male predominance, and was managed predominantly with endourological procedures. Procedure distribution varied markedly according to stone location and size. The purpose of these analyses was not to establish the already recognized principle that stone anatomy influences treatment choice, but to quantify how these patterns were expressed within the surgical case mix of this regional referral service.

The demographic findings are consistent with the broader epidemiology of stone disease. Contemporary pooled data estimate urolithiasis prevalence at approximately one in 10 adults globally, with substantial geographic and methodological heterogeneity [1], and epidemiologic reviews continue to describe a higher burden among men in many populations [2]. Brazilian public-hospital data have also shown that stone disease generates sustained demand across working-age and older adults [4]. Because the present sample was restricted to surgically treated patients at a referral hospital, however, the observed age and sex distributions should not be interpreted as population prevalence or incidence. The geographic distribution also reflects the referral character of the service. Vitoria, Serra, Vila Velha, and Cariacica together accounted for 67.0% of the cohort, while patients were also referred from municipalities outside the metropolitan area. These data describe the referral catchment of the surgically treated cohort and should not be interpreted as municipality-specific incidence or access rates because population denominators and referral probabilities were not assessed.

The predominance of ureteroscopy in this cohort is particularly relevant in the Brazilian context. A national survey found that rigid ureteroscopy was widely performed by Brazilian urologists, whereas availability and practical experience for flexible ureteroscopy and percutaneous nephrolithotomy were less uniform, with equipment and infrastructure identified as barriers in some regions [5]. In the present service, more than nine in 10 patients were managed with a ureteroscopic procedure. This pattern has practical implications for service planning, including the availability of endourological equipment and trained personnel, but it should not be interpreted as a direct measure of resource adequacy or treatment quality.

Procedure distribution varied markedly according to stone location. Flexible ureterorenoscopy predominated in proximal ureteral disease, rigid ureteroscopy in distal ureteral disease, and percutaneous nephrolithotomy in intrarenal disease. This pattern is clinically coherent with contemporary treatment frameworks, which incorporate anatomic access, stone burden, collecting system characteristics, equipment, and surgeon expertise into procedural selection [6-9]. The very large effect size should therefore be interpreted as a quantitative description of local practice and case mix rather than evidence of a novel causal mechanism or proof of guideline adherence. The predominance of ureteral over intrarenal stones in this cohort (417 proximal ureteral, 160 distal ureteral, and 42 intrarenal cases) should also be considered a characteristic of this referral population rather than a population-level distribution of urolithiasis.

Stone size provided complementary information. Ureteroscopic techniques predominated in the categories up to 20 mm, whereas percutaneous nephrolithotomy was the most frequent procedure among stones >20 mm. Among the relatively small intrarenal subgroup with complete size data (n = 40), 34 patients had stones >20 mm. This distribution may partly account for the observed association between intrarenal location and percutaneous treatment. The pattern is clinically consistent with contemporary guidance regarding larger renal stones and the use of ureteroscopy for ureteral and selected renal stones [6-8]. Nonetheless, size alone does not determine treatment, and the present dataset lacked several variables needed to judge appropriateness at the individual-patient level.

The anesthetic profile was notable because spinal anesthesia, alone or combined with sedation, was used in 89.6% of the cohort. This finding should be interpreted as a description of local perioperative practice rather than evidence favoring regional over general anesthesia. Procedure-specific anesthetic indications, operative duration, conversion to general anesthesia, postoperative pain, recovery time, and anesthetic adverse events were not systematically captured. These missing outcomes preclude comparative conclusions but identify an area that could be examined prospectively in high-volume endourological services.

A double-J ureteral stent was used in 71.8% of patients. Because the database did not systematically record the indication or timing of stent placement, this finding should be interpreted as a service-level utilization measure rather than an assessment of guideline adherence or appropriateness of individual stent use.

Several features strengthen the study. All eligible surgically treated cases identified during the study period were included rather than sampled selectively, producing a relatively large single-center cohort. The dataset integrated demographics, geographic origin, stone characteristics, procedure type, anesthesia, laterality, stent use, and multiple-procedure cases. In addition, the main relationships were expressed with effect-size estimates rather than p-values alone, which helps distinguish statistical significance from the magnitude of association.

The study also has important limitations. Its retrospective, single-center design makes the analysis dependent on routine documentation and limits external generalizability. Stone size was undocumented in 33 patients and location in five. Stone composition, Hounsfield units, volumetric burden, detailed collecting system anatomy, preoperative infection, renal function, comorbidity burden, surgeon-level factors, operative time, complications, length of stay, stone-free status, readmission, reintervention, and long-term outcomes were not systematically available. These missing variables precluded a meaningful multivariable model and prevented causal or comparative-effectiveness interpretation. The seven-month study period may also be affected by temporal and referral-pattern variability. Twenty-two patients underwent more than one procedure during the same operative session: 17 underwent bilateral flexible ureterorenoscopy, and five underwent percutaneous nephrolithotomy with complementary flexible ureterorenoscopy. Each patient contributed a single procedure category to the contingency analyses. In bilateral stone disease, using the larger stone for stone-size classification may modestly shift the size distribution toward greater stone burden, although the rule was applied consistently to preserve patient-level independence.

Despite these limitations, the study establishes a useful institutional baseline for real-world stone surgery. The findings may inform local planning, record standardization, and prospective outcome collection. A logical next step is a multicenter prospective study using standardized stone-burden variables and postoperative outcomes, including complications, stone-free status, length of stay, readmission, reintervention, and patient-reported measures. Such work would move beyond procedure distribution toward assessment of effectiveness, quality, and generalizability across different Brazilian practice settings.

## Conclusions

Surgical treatment of urolithiasis at this Brazilian referral hospital was characterized by extensive use of minimally invasive endourological procedures and by marked variation in procedure distribution according to stone location and size. The findings define the service's real-world surgical case mix and quantify the distribution of procedures across stone location and size categories.

These data provide a practical baseline for service planning and prospective quality assessment. Future multicenter studies incorporating clinical complexity, perioperative complications, stone-free outcomes, length of stay, readmission, reintervention, and patient-reported outcomes are needed to determine the effectiveness and generalizability of these treatment patterns.

## References

[1] Vera-Ponce VJ, Sanchez-Tamay NM, Ballena-Caicedo J, Zuzunaga-Montoya FE, De Carrillo CI, Poemape Mestanza RL (2025). Global prevalence of urolithiasis: a meta-analysis accounting for methodological heterogeneity. Front Urol.

[2] Abufaraj M, Al Karmi J, Yang L (2022). Prevalence and trends of urolithiasis among adults. Curr Opin Urol.

[3] Carvalho M, Matos AC, Santos DR (2025). Brazilian guidelines on evaluation and clinical management of nephrolithiasis: Brazilian Society of Nephrology. J Bras Nefrol.

[4] Mello MF, Marchini GS, Câmara C (2016). A large 15-year database analysis on the influence of age, gender, race, obesity and income on hospitalization rates due to stone disease. Int Braz J Urol.

[5] Astolfi RH, Carrera R, Gattas N, Bertolla R, Sepulveda F, Reggio E, Meller AE (2020). Current scenario of endourological treatment of kidney stones in brazil: results of a national survey. Int Braz J Urol.

[6] (2026). European Association of Urology: EAU guidelines on urolithiasis. https://uroweb.org/guidelines/urolithiasis.

[7] Akram M, Jahrreiss V, Skolarikos A (2024). Urological guidelines for kidney stones: overview and comprehensive update. J Clin Med.

[8] Geraghty RM, Davis NF, Tzelves L (2023). Best practice in interventional management of urolithiasis: an update from the European Association of Urology guidelines panel for urolithiasis 2022. Eur Urol Focus.

[9] Assimos D, Krambeck A, Miller NL (2016). Surgical management of stones: American Urological Association/Endourological Society guideline, part I. J Urol.

[10] von Elm E, Altman DG, Egger M, Pocock SJ, Gøtzsche PC, Vandenbroucke JP (2007). The Strengthening the Reporting of Observational Studies in Epidemiology (STROBE) statement: guidelines for reporting observational studies. Lancet.

